# Prenatal Screening of Chromosomal Anomalies Using Genome-Wide or Target Cell-Free DNA: Preferences and Satisfaction of Pregnant Women

**DOI:** 10.3390/jcm13164888

**Published:** 2024-08-19

**Authors:** Victoria Ardiles-Ruesjas, Roser Viñals, Montse Pauta, Irene Madrigal, Antoni Borrell

**Affiliations:** 1 BCNatal-Barcelona Centre for Maternal-Fetal and Neonatal Medicine, Hospital Clinic de Barcelona, 08036 Barcelona, Spain; ardiles@recerca.clinic.cat (V.A.-R.); rvinals@clinic.cat (R.V.); montse_pauta@hotmail.com (M.P.); 2 Fundació de Recerca Clínic Barcelona-Institut d’Investigacions Biomèdiques August Pi i Sunyer (FRCB-IDIBAPS), 08036 Barcelona, Spain; 3 Biomedical Diagnosis Center, Hospital Clinic de Barcelona, 08036 Barcelona, Spain; imadbajo@clinic.cat; 4 Medical School, Universitat de Barcelona, 08907 Barcelona, Spain

**Keywords:** screening, genome-wide cell-free DNA, non-invasive prenatal testing, decision-making, regret, preferences

## Abstract

**Background/Objectives**: Cell-free DNA (cfDNA) is a non-invasive prenatal test used to screen for common trisomies (target cfDNA) that can be expanded to assess all autosomal chromosomes (genome-wide cfDNA). As cfDNA testing gains popularity, it is crucial to examine the factors influencing the decision-making process of pregnant individuals when choosing between these two approaches. **Methods**: In this prospective cohort study, 190 individuals undergoing cfDNA testing for aneuploidy screening, according to the current screening protocol, were allowed to make their own choice between target and genome-wide cfDNA testing. They were asked to complete a first survey at 11–13 weeks, designed to explore their characteristics, preferences, and satisfaction with the prenatal genetic counseling session, as well as a Decisional Conflict Scale. A postnatal survey was administered three months after delivery, including the Decisional Regret Scale and two open questions. **Results**: 84% of participants opted for genome-wide cfDNA. However, 17% found the decision challenging, and 14% felt that the results might increase anxiety. No significant differences in participant characteristics were found when comparing decisions between genome-wide and target cfDNA. However, significant differences were observed regarding ethnicity (*p* = <0.001), educational level (*p* = 0.029), previous cfDNA experience (*p* = 0.004), and having sufficient information when comparing termination options (*p* = 0.002). After delivery, only 4% would have changed their decision. **Conclusions**: Individuals, regardless of their characteristics, prefer genome-wide cfDNA; however, the complexity of the results necessitates enhanced genetic education for prenatal care clinicians.

## 1. Introduction

Strategies for aneuploidy screening have been in place since the 1970s, and have evolved from the advanced maternal age criterion to the first trimester combined test and cell-free DNA (cfDNA) testing. The combined test modifies the specific trisomy risk corresponding to maternal age with likelihood ratios derived from two biochemical and one ultrasound marker [1]. This strategy could detect 90% of common trisomies, with a 5% false-positive rate. Target cfDNA testing is also focused on common trisomies, with detection rising to 96–99% and false-positive rates declining below 0.5% [2]. cfDNA testing is clearly a better screening method, but its current cost jeopardizes a wider implementation to the entire pregnant population.

The extension of the target cfDNA test to other chromosomes and large copy number variations (deletions and duplications) is controversial. It is commonly known as “genome-wide cfDNA” and can detect a variable proportion of the chromosomal anomalies that can be diagnosed with a karyotype. Mark Pertile achieved detection rates of 96% for autosomal trisomies and 74% for large deletions/duplications [3], while the TRIDENT-2 study demonstrated 6% and 32% positive predictive values in the general pregnant population, respectively [4]. However, many centers have expressed their opposition to genome-wide cfDNA. Hence, Jani et al. [5] argued that: (a) there is uncertainty after a positive atypical trisomy result, either confirmed or not; (b) false-positives and invasive testing rates increase; (c) it is not recommended by scientific societies; (d) it raises ethical and legal challenges regarding how to counsel; and (e) it is a violation of WHO screening principles. In reply, the Dutch group [6] argued that (a) 78% of women prefer genome-wide cfDNA when it is offered; (b) atypical trisomies carry an increased risk (23–45%) of adverse pregnancy outcomes; (c) 39% of autosomal trisomies are not detected by ultrasound; (d) genome-wide cfDNA can detect maternal malignancies; (e) there is only a 0.4% excess of invasive testing; and (f) clinical geneticists counsel all atypical results.

Nowadays, screening protocols cannot be supported only with scientific evidence and expert opinions without considering the perspectives, preferences, and opinions of pregnant women. There is much data about women’s preferences in decision-making between cfDNA and invasive procedures, yet scarce data between target and genome-wide cfDNA. In a previous study of our group, pregnant women with a high risk of common trisomies were asked if they would prefer cfDNA or invasive testing as a further test; half of the pregnant women opted for cfDNA testing [7]. The main reason to choose cfDNA was to avoid the risk of pregnancy loss. Women using assisted reproductive techniques and those of Latin American origin preferred cfDNA testing, while nonreligious women and those with a favorable opinion on the termination of pregnancy preferred invasive testing.

Nowadays, cfDNA testing is gaining popularity due to its non-invasive nature and high sensitivity. The genome-wide approach has been questioned by some academic centers, claiming that data on its clinical implementation are scarce [5]. Hence, this study was conducted to add more data on women’s preferences and satisfaction. The main aim of the present study was to compare two groups of women, those who decided to undergo target cfDNA and those who preferred genome-wide cfDNA testing, regarding (a) women and pregnancy characteristics; (b) termination options; (c) preferences and satisfaction with genetic counseling; (d) decisional conflicts; and (e) decisional regrets.

## 2. Methods

### 2.1. Study Design and Sample

This was a prospective cohort study utilizing a mixed-methods survey that included both structured questionnaires and open-ended questions. Pregnant women undergoing cfDNA testing for aneuploidy screening from March 2022 to April 2023 were asked to participate. According to the current aneuploidy screening protocol in Catalonia [8], pregnant women with a high risk (1/11–1/250) at the first trimester combined test and those with a previous trisomy 21, 18, or 13 are entitled to choose between (a) quantitative fluorescent polymerase chain reaction (QF-PCR) with chromosomal microarray analysis (CMA) in chorionic villi or amniotic fluid; (b) target cfDNA testing; and (c) no further tests. Women and partners were seen by a prenatal geneticist or a fetal medicine specialist for pre-test genetic counseling. Women with an intermediate risk (1/251–1/1100) at the combined test were only offered cfDNA testing by a nurse specialized in genetics. According to the current Catalan protocol, in both situations, the cfDNA testing routinely offered is the targeted test, confined to trisomies 21, 18, and 13. The cfDNA test is fully funded by the government through the public health system, and it is important to note that the same kit can be used to analyze either three specific chromosomes or all the chromosomes. The exclusion criteria of the study were the following: (a) fetuses with structural anomalies at the early anomaly scan; (b) fetuses with an increased nuchal translucency above the 99th percentile at 11–13 weeks; and (c) family history of genetic diseases.

The genetics specialized nurse (R.V.) or the prenatal geneticist (A.B.) offered the extended test to all chromosomes, known as “genome-wide cfDNA”, to women undergoing target cfDNA testing according to the current Catalan protocol. Given that its resolution is 7 Mb, the detectable spectrum of anomalies, but not its sensitivity, is coincidental with that of the karyotype and includes aneuploidies, deletions, and duplications. The advantages and disadvantages of genome-wide cfDNA were discussed with the women, ideally together with their partners. A second informed consent process was carried out, and another document was signed by the women. The reasons for specific exclusion in this second part were (a) minors and (b) those who did not have sufficient proficiency in Spanish or Catalan to read and understand the questionnaire.

### 2.2. Prenatal Survey

Pregnant women were requested to complete a questionnaire encompassing: (a) the demographic and pregnancy characteristics; (b) the options for pregnancy termination; (c) a 15-item Likert-type questionnaire focusing on aspects related to satisfaction with the genetic consultation; and (d) the O’Connor’s Decisional Conflict Scale (DCS) [9,10], consisting of 16 questions, aimed to identify any conflicts that women may encounter in making healthcare decisions.

Regarding the psychometric properties of the latter two questionnaires, it should be pointed out firstly that, since there is no specific tool for prenatal genetic consultation, the existing Spanish version [11] of the Genetic Counseling Outcome Scale (GCOS-24) [11] was thoughtfully curated into a new questionnaire. Secondly, the Spanish version of the DCS had already been validated among Spanish-speaking populations, demonstrating commendable psychometric properties: reliability, measured by Cronbach’s α, surpassing 0.87, along with robust correlations among its subscales, affirming its construct validity [12].

### 2.3. Sequencing cfDNA and Confirmatory Diagnostic Procedures

After completing the survey, a peripheral venous blood sample (10 mL) was obtained from each pregnant woman in a Streck blood collection tube and sent to the molecular lab the same day. cfDNA testing was performed using next-generation sequencing (NGS)-based technology (VeriSeq NIPT Solution v2) (Illumina Inc., San Diego, CA, USA). The protocol was performed according to the supplier’s specifications and whole-genome surface sequencing was carried out on the NextSeq500Dx sequencer (Illumina Inc., San Diego, CA, USA). Data were analyzed using VeriSeq NIPT Assay Software v2 (Illumina Inc., San Diego, CA, USA), resulting in an “Aneuploidy detected” or “No aneuploidy detected” result for each of the tested chromosomes. When parents opted for the targeted test, a filter was applied to reveal only the results of chromosomes 21, 18, and 13; if parents opted for the genome-wide approach, the analysis of all chromosomes was reported, including rare trisomies, sex aneuploidies, and deletions or duplications larger than 7 Mb.

When a high risk of a chromosomal anomaly was displayed, diagnostic confirmation was offered through invasive diagnostic testing by means of (a) chorionic villus sampling for trisomy 21 cases and for trisomies 13 and 18 with ultrasound anomalies at 10–13 weeks, or (b) amniocentesis for other anomalies from week 16 onwards. A QF-PCR was applied first, allowing the diagnosis of trisomy 21, 18, or 13, and was followed by a conventional karyotype if trisomy 21 or 13 was found. In normal QF-PCR results, CMA was performed. In the case of a high risk of trisomy of an imprinted chromosome, uniparental disomy studies were performed using samples from both parents.

### 2.4. Postnatal Survey

Three months after delivery, a follow-up survey was sent to all participants encompassing a five-item Likert scale of the Spanish version of the Decision Regret Scale (DRS), validated in the Spanish population [13], together with two open questions: (a) whether they would change their decision and why; and (b) whether they felt more in control. The integration of both quantitative and qualitative methods with open questions provides a better understanding of the data, allowing for a more holistic interpretation of the findings.

### 2.5. Data Analysis

Regarding the first prenatal questionnaire on maternal and pregnancy characteristics, quantitative variables were expressed as mean and standard deviation (SD), while qualitative variables were expressed in absolute numbers and proportions. To optimize the evaluation, any missing results were excluded from consideration, and the total count of such instances was systematically recorded. A multivariate exploratory data analysis was conducted to evaluate the significance of the differences in women and pregnancy characteristics between women choosing the targeted or the genome-wide approach by using Fisher’s exact test, which is typically used for categorical data and especially when the sample sizes are small or data are scarce. Women and pregnancy characteristics were also compared between women with different termination options using the same test. For quantitative variables—maternal age, gestational age, fetal fraction, and nuchal translucency—the assumption of normality was first evaluated using the Shapiro–Wilk test. Since none of these variables followed a normal distribution, the non-parametric Mann–Whitney U test was employed to compare differences between the two independent groups. The results of the preferences and satisfaction questionnaire and the DCS were assessed separately and plotted in a graph. Differences between the two study groups were also assessed with Fisher’s statistical test. Positive results, either in target or extended tests, were followed up to delivery and hospital discharge. No-calls were also followed.

Regarding the postnatal survey, a five-question Decisional Regret Scale was filled out by the women, and results were compared between the two study groups, also using Fisher’s test. Furthermore, exploratory data analysis was conducted to summarize the answers to the two open-ended questions. The written responses were incorporated into the database and amounted to 42 short comments overall. Subsequently, a thematic content analysis was conducted manually. The process consisted of the following steps: a first set of readings to comprehend the gathered information; the creation of codes without interpretation of excerpts of phrases; the formation of categories by consolidating various codes; and the grouping of these categories into higher-level categories. Subsequently, the main ideas were summarized and presented in the Section 3. The responses were anonymized with the letter C and the case number (e.g., C-33).

## 3. Results

### 3.1. Women and Pregnancy Characteristics

There were 190 women enrolled in the study. The mean maternal age was 37.1 years (SD ± 4.1), while the mean gestational age was 12.8 weeks (SD ± 1.0), with no significant differences observed (Table 1). Most of the pregnant women were of European (72%) and Latin American (21%) origin, had a college education (78%), and faced pregnancy with a partner (96%). Among participants, 60% had experienced a previous pregnancy, 36% reported having at least one child alive, and 29% had suffered a pregnancy loss. The present pregnancy was conceived by in vitro fertilization in 26% of women, 12% had already undergone a private cfDNA test, while 15% had had other genetic tests, such as preimplantation genetic testing, microarrays, or NGS (Table 1). In the vast majority of pregnancies, the first-trimester scan was performed the same day the survey was filled out, and the mean nuchal translucency was 2 mm (SD ± 0.6), and no significant differences were found between the groups choosing the targeted or genome-wide test (Table 1).

Regarding the reason for cfDNA testing, 12% of the pregnant women exhibited a high risk of trisomy 21 (1/11–1/250), 78% had an intermediate risk (1/251–1/1100), and 10% had a low risk (<1/1100). Regarding trisomy 18, 98% of participants were categorized as low-risk, with 1% each falling into the intermediate- and high-risk categories. Concerning trisomy 13, 99% of participants were deemed low-risk, while the remaining 1% were classified as high-risk. One woman received a high risk of trisomies 18 and 13 and a low risk of trisomy 21; another two women had an intermediate risk of trisomy 18, a low risk of trisomy 13, and a high or intermediate risk of trisomy 21. When asked about which cfDNA approach they preferred, 159 women (84%) chose a genome-wide analysis, while 31 (16%) preferred the target analysis (Table 1). No differences were observed in demographic and pregnancy characteristics between women opting for either the target cfDNA or genome-wide cfDNA method (Table 1).

### 3.2. Options for Pregnancy Termination

Regarding the couple’s options when facing a genetic anomaly, 79 (42%) of participants stated that they considered the termination of pregnancy an option, while 17 (9%) stated they would never terminate the pregnancy under any circumstances, and 92 (49%) expressed uncertainty about what action to take (Table 1). In the stratified analysis of demographic and pregnancy characteristics according to the couple’s termination option, four variables presented significant differences: educational level, ethnicity, previous cfDNA, and having sufficient information (Table 2). In greater detail, among college students, 68 considered termination, 9 were against it, and 70 were unsure about what to do. Similarly, within the European ethnicity group, 69 participants would consider termination, 3 were against it, and 64 were unsure about what to do. Among Latin American individuals, 10 considered termination, 12 were against it, and 17 were uncertain. Ultimately, among women who felt they had received sufficient information, 77 would consider termination, 12 were against it, and 82 were uncertain (Table 2).

### 3.3. Women’s Preferences and Satisfaction

**Figure 1:**
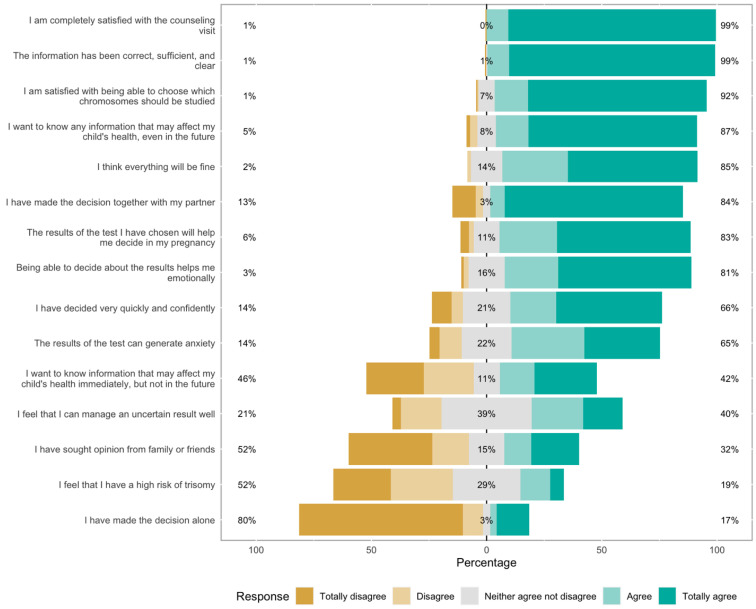
Perspectives and satisfaction questionnaire after the genetic counseling session of the entire study group.

Half (52%) of the women had the perception of being at risk of trisomies, while 19% did not. Two-thirds (66%) of women decided on the cfDNA approach very quickly and confidently, 81% felt that being able to decide about the test may help them emotionally, although 65% of them believed that the results could produce anxiety (Figure 1). 84% percent of individuals decided on the cfDNA approach together with their partners, while 16% chose it on their own. Furthermore, 52% of them did not ask the opinion of their family and friends, while 32% did (Figure 1). Ultimately, 85% of women were confident that everything about pregnancy would proceed favorably (Figure 1). Concerning the genetic counseling session, 99% of women stated that they were completely satisfied (Figure 1), and 92% strongly agreed with being able to choose which chromosomes should be studied at cfDNA testing, while 8% expressed some doubts (Figure 1).

Significant differences between the target and genome-wide cfDNA groups were observed in five of the items in the perspectives and satisfaction questionnaire (Table 3). Firstly, women feeling that they could manage an uncertain result accounted for 43% of women in the genome-wide group and 26% in the target group (Table 3). Regarding the assumption that the test results would help with their pregnancy decision-making, it was adopted by 85% of the genome-wide group and 71% of the target group (Table 3). Women who wished to know any potential impact that could affect the health of their fetuses, even in the future, accounted for 93% of the genome-wide group and 59% of the target group (*p* < 0.001) (Table 3).

### 3.4. The Decisional Conflict Scale

**Figure 2:**
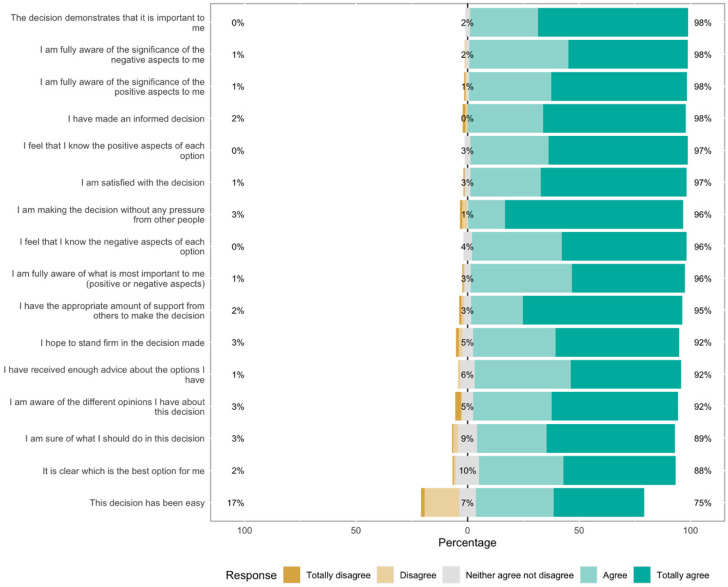
Decisional Conflict Scale (DCS) of the entire study group.

The DCS results support several findings obtained from the perspectives and satisfaction questionnaire. Respondents either agreed or strongly agreed with 15 of the 16 items in the questionnaire. However, one statement, ‘The decision has been easy’, exhibits some variation in opinions: while 75% agreed, 17% disagreed (Figure 2). The only item in which significant differences were found between the target and the genome-wide groups was expressing satisfaction with the decision: 98% in the genome-wide group and 90% in the target group (*p* = 0.036) (Table 3).

### 3.5. cfDNA Results and No-Calls

The mean fetal fraction was 11.7% (SD ± 4.3) (Table 1). There were no cases in which a conclusive result could not be achieved. Only three individuals (1.6%) received high-risk results: two for Klinefelter syndrome, which we confirmed at diagnostic testing, and a 41.7 Mb deletion (del17q21.12q32.2) that was not confirmed. The three babies were successfully delivered.

### 3.6. Decisional Regret Scale

**Figure 3:**
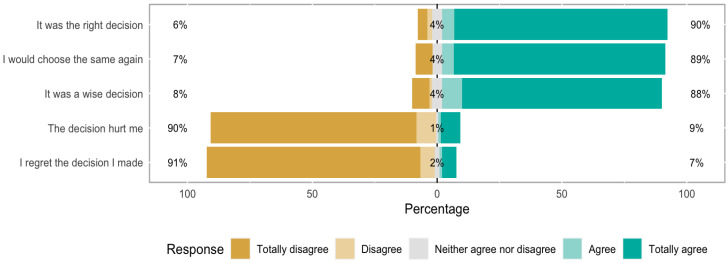
Decisional Regret Scale (DRS) of the entire study group.

Among the 190 initial participants, 105 responded to the postnatal survey, which included the DRS and two open questions. Regarding the DRS results (Figure 3), highlighting the decision-making process, most participants (90%) felt that it was a good decision, while 91% strongly disagreed with the premise of regretting the decision. Similarly, 89% believed they would make the same decision again, and 89% thought it was a wise decision. Finally, 90% disagreed that the decision caused them pain (Figure 3). No significant differences were found between the target and genome-wide groups.

Finally, in relation to the two open questions, only four (4%) respondents stated that they would alter their decision regarding the cfDNA approach adopted: two from the target group and two from the genome-wide group. Surprisingly, nine women (8.6%) wrongly stated having taken the target test instead of the genome-wide test, despite receiving results from the latter. Moreover, 83% of pregnant participants reported feeling more in control, while 17% did not (Table 3). Significant differences were not found between the groups.

### 3.7. Postnatal Open Questions

A total of 42 observations were compiled when asked the reason for their choice. Individuals who would not change their decision and felt more in control attributed this to having access to more data. They described positive emotions, such as an increased reassurance associated with being able to make their choice. For instance, one participant stated, “By having more data about the presence or absence of significant illnesses in my daughter, I was able to decide what to do with my pregnancy (whether to go on with it or not). Additionally, it has given me much more mental peace and I believe it has positively affected my pregnancy” (C-4).

Some women emphasized the support of the healthcare team in facilitating their decision-making process and providing information about the test: “They have always asked for my opinion first and explained what the test entails” (C-115). Additionally, there were concerns raised about the socio–economic aspect of access to such tests, with one participant noting, “(…) this option is only available to people who can afford it, which I don’t think is fair and creates a social division” (C-5), although, in this study, all tests were provided at no cost by the National Health System.

Some other participants stated that they felt more reassured than being in control, and some argued that adequate support and information are crucial to providing more control. “Control over gestation is not provided by a diagnosis, but by an adequate support that includes good information, which was not the case” (C-166) was argued by an individual who received a positive result for a genetic anomaly in the genome-wide test. Few participants expressed dissatisfaction with the requested test. A pregnant individual with a false-positive result expressed doubts about the sense of control and the validity of the results, stating, “Yes and no. I had a problem with the genome-wide test because it was a false-positive” (C-188).

## 4. Discussion

### 4.1. Main Findings

The main findings of this study were the following: (a) 84% of women opted for genome-wide cfDNA when target cfDNA is indicated for high or intermediate risks at the combined test; (b) 93% of those choosing the genome-wide analysis argued that they wished to know as much as possible about their child’s health; (c) termination of pregnancy was a prospective option for 42% of the pregnant women, mainly Caucasian, with a college education, and satisfied with the genetic counseling session; (d) 99% of women were satisfied with the option of being able to decide the cfDNA approach and with the genetic counseling session, although 14% felt that results may increase anxiety; (e) 17% thought that this decision had not been easy; and (f) after delivery, only 4% of participants would have changed their decision.

### 4.2. Comparison with the Literature

There are scarce data on women’s preferences, decision-making processes, satisfaction, and regrets concerning genome-wide cfDNA testing against target cfDNA uptake in front of the combined test or invasive testing. In the Netherlands, genome-wide cfDNA testing is offered to all pregnant women as part of the nationwide screening program TRIDENT-2. A prospective survey was conducted during the years 2017–2018 with 473 respondents, 77% of whom chose genome-wide cfDNA and 22% target cfDNA [14]. Univariate logistic regression analysis revealed that the variables age, education level, country of origin, religion, parity, health literacy, and gestational age were not significantly associated with the decision for either genome-wide or target cfDNA. This could mean that, regardless of their characteristics, pregnant individuals usually desire more information regarding their future child’s health status. Similarly, in our series, the proportion of genome-wide/target cfDNA was 84%/16%, and no differences were observed in women or pregnancy characteristics between the two groups.

In the prospective Dutch study, the main reasons to choose genome-wide cfDNA were: ‘wanting as much information as possible regarding the child’s health’ (39%), ‘to be prepared for everything’ (24%), and ‘making optimal use of the test’s abilities’ (e.g., no additional costs and the analysis is being performed anyway) (14%) [14]. For 4.8%, receiving information about her own health was a reason to choose genome-wide cfDNA. The main reasons of the 86 respondents who chose against genome-wide cfDNA were: ‘avoiding uncertain results/outcomes’ (34%) and ‘not wanting to unnecessarily worry’ (33%). The reasons pointed out in our study are similar to those compiled in the Dutch study, specifically: ‘wanting as much information as possible’ and ‘to be prepared for everything’. Nevertheless, it seems that our population was more concerned with the quality of assistance and care than with the diagnostic results themselves.

In our series, we found that European, highly educated women satisfied with the counseling process were more prone to terminate the pregnancy of an affected fetus. In a survey comparing perspectives and decision-making between women undergoing genome-wide cfDNA testing in the Netherlands (NL) and Belgium (BE), significant differences were found in the intention to terminate the pregnancy in the case of confirmed trisomy 21 (NL: 51% vs. BE: 62%). Differences were also seen in considering trisomy 21 a severe condition (NL: 64% vs. BE: 81%, *p* < 0.001) and appear to be explained by being less positive about the quality of care and support for children with trisomy 21 (BE: 23% vs. NL: 62%) [15].

In our study, 99% of women stated that they were completely satisfied, and 92% strongly agreed with being able to choose between target and genome-wide cfDNA. Among the respondents in the prospective Dutch study, 99.2% were glad to have been offered cfDNA and 99.6% reported that, in retrospect, they would make the same choice [14]. Nearly all (98.9%) of the respondents who elected to have genome-wide cfDNA were glad that cfDNA could be used to detect findings other than trisomies 21, 18, and 13, compared with 39% of respondents who chose target cfDNA. A second Dutch survey was performed retrospectively among women in whom the genome-wide cfDNA test revealed a rare trisomy, a segmental imbalance, or a complex karyotype named “additional findings” [16]. In this study, almost all women (98%) reported having had a pre-test counseling session with their obstetric caregiver about prenatal screening; however, about half of these women (54%) believed that the caregiver did not pay enough attention to these additional findings during counseling.

Regarding decisional conflicts, in our series, 75% of women agreed that making this choice between both approaches was an easy decision, and 17% disagreed, with no significant differences between both groups. Among respondents in the Dutch prospective study who chose genome-wide cfDNA, 18% found that it was a (somewhat) difficult choice, compared with 40% of respondents who chose target cfDNA (*p* < 0.001) [14]. In the Dutch retrospective study, a slight majority of women (59%) in whom the genome-wide cfDNA revealed an additional finding did not find it difficult to choose between the two cfDNA approaches, 29% did find it difficult to make a decision, and 13% had a neutral opinion [16].

Finally, regarding decisional regrets in our study, only 4% of respondents would have changed their decision when asked three months after delivery. In the prospective Dutch study, 95% of women after receiving their low-risk result felt reassured, and 97% did not regret testing, while one (17%) woman, after receiving a high-risk result, reported regretting that she chose cfDNA testing [14].

### 4.3. Clinical Application

It appears that women in Southern Europe have the same interest as Central Western European women in making their own decisions once a pre-test counseling session is provided. Comparisons were hampered by differences in prenatal screening methods, since the Netherlands and Belgium offer cfDNA testing to the entire pregnant population, while in our setting cfDNA is only provided in high- and intermediate-risk pregnancies.

Interestingly enough, the clinical application of genome-wide cfDNA is facilitated by the fact that one of the main commercial companies supplies a kit that includes all chromosomes, even for a target cfDNA study, and subsequently filters the results for non-desired chromosomes. On the other hand, it is crucial to consider that access to cfDNA varies across different populations, even within the same country. For instance, an Australian study found that cfDNA-indicated testing was significantly more common in metropolitan areas, not due to geographical factors but because of underlying socioeconomic disparities [17].

The main drawback of the implementation of genome-wide cfDNA is that the pre-test counseling session is more complicated, as rare trisomies may affect the pregnancy even when they are not found in the fetus [18]. Positive predictive values and clinical utility must be addressed for each of the chromosomal anomaly types: typical trisomies, atypical trisomies, sex aneuploidies, and segmental imbalances. Specifically, in atypical trisomies, it should be highlighted that although the positive predictive value is very low, the cfDNA clinical utility has been demonstrated in an increasing number of studies [3,5,14,15,17]. The increasing complexity of counseling may make prenatal care clinicians feel uncomfortable when discussing the possible results with their patients, regardless of their medical literacy levels, as this has a direct impact on the decisions pregnant women will take going forward. Another drawback of genome-wide cfDNA testing is related to the increased burden of false-positives. According to the Belgian and Dutch experience, the screen-positive rate for atypical trisomies and deletions/duplications (0.35%) is lower than that for typical trisomies (0.5%), although the false-positive rate is higher (0.27%) for the former as compared to the latter (0.03%). However, those rates are clearly inferior to the observed rates at the combined test (0.5% screen-positive rate and 0.4% false-positive rate) [4,19], and most of them are not entirely false-positive cases because an adverse outcome may be expected in confined placental mosaicism.

### 4.4. Limitations and Strengths

Our study is not without limitations. Firstly, there is a noticeable tendency toward acquiescence in the responses to the DCS questionnaire, with women predominantly agreeing with the items regardless of their content. We believe that this may be a bias stemming from the length of the test, which could have led to a fatigue effect among the participants. Secondly, 8.6% of the women wrongly recalled having taken the target test instead of the genome-wide test three months after delivery. The underlying issue may be attributed to a lack of clear understanding among the participants regarding the specific test they were undergoing. This connects to the fact that women’s health literacy has not been studied, and, therefore, insights into their actual level of knowledge have not been provided, and existing knowledge gaps would have been revealed. Thirdly, by restricting the inclusion to Spanish and Catalan-speaking women, the study sample is homogeneous and does not accurately reflect the diverse pregnant population of our center, which limits the generalizability of the findings. Finally, it seems necessary that further studies should have questionnaire versions in Urdu, Panjabi, Mandarin, and Darija. On the other hand, a strength of our study is that it is one of the first studies on women’s perceptions, preferences, and satisfaction conducted in Southern Europe, given that most of them have been performed in the UK and the Netherlands. Another novelty of our study is that genome-wide cfDNA is offered to individuals with high and intermediate risks, rather than to the general pregnant population, and therefore the conclusions of our study can be more valuable when applied to these risk groups.

## Figures and Tables

**Table 1:** Women and pregnancy characteristics, stratified according to the cfDNA approach chosen by the women, target or genome-wide. For continuous variables: mean, standard deviation (SD), and missing values, and for categorical variables: N (%).

	Total (n = 190)	Target (n = 31)	Genome-Wide (n = 159)		
	Mean (SD)	Mean (SD)	Mean (SD)	Missing	Overall *p*
**Age (years)**	37.1 (4.1)	37.6 (3.2)	36.9 (4.3)	0	0.433
**Gestational age (weeks)**	12.8 (1)	12.9 (0.6)	12.8 (1)	8	0.368
**Nuchal translucency (mm)**	2 (0.6)	2 (0.4)	2 (0.6)	14	0.584
**Fetal fraction (%)**	11.7 (4.3)	10.2 (3.7)	11.2 (4.4)	2	0.204
	**N (%)**	**Target** **(n = 31)**	**Genome-Wide (n = 159)**	**Overall *p***	
**Education level**	(missing 1)			0.060	
Up to secondary	24 (13%)	3 (9.7%)	21 (13%)		
Professional training	17 (9%)	7 (23%)	10 (6.3%)		
University	148 (78%)	21 (68%)	127 (80%)		
**Ethnicity**	(missing 0)			0.167	
European	137 (72%)	22 (71%)	115 (72%)		
Latin American	40 (21%)	7 (23%)	33 (21%)		
Asian	4 (2%)	2 (6.5%)	2 (1.3%)		
Other	9 (5%)	0 (0%)	9 (5.7%)		
**Marital status**	(missing 0)			0.491	
In a relationship	183 (96%)	30 (97%)	153 (96%)		
Single parent	4 (2%)	0 (0%)	4 (2.5%)		
Separated	3 (2%)	1 (3.2%)	2 (1.3%)		
**Previous pregnancy**	(missing 1)			0.630	
No	75 (40%)	14 (45%)	61 (39%)		
Yes	114 (60%)	17 (55%)	97 (61%)		
**Previous pregnancy loss**	(missing 1)			0.877	
No	135 (68%)	23 (74%)	112 (71%)		
Yes	54 (32%)	8 (26%)	46 (29%)		
**Children**	(missing 1)			0.641	
0	120 (63%)	21 (68%)	99 (63%)		
1	55 (29%)	7 (23%)	48 (30%)		
2 or more	14 (7%)	3 (9.7%)	11 (7%)		
**In vitro fertilization**	(missing 0)			0.261	
No	141 (74%)	20 (65%)	121 (76%)		
Yes	49 (26%)	11 (36%)	38 (24%)		
**Sufficient information**	(missing 0)			1.000	
No	17 (9%)	3 (9.7%)	14 (8.8%)		
Yes	173 (91%)	28 (90%)	145 (91%)		
**Previous cfDNA**	(missing 0)			0.380	
No	167 (88%)	29 (94%)	138 (87%)		
Yes	23 (12%)	2 (6.5%)	21 (13%)		
**Other previous genetic tests**	(missing 0)			0.176	
No	162 (85%)	24 (77%)	138 (87%)		
Yes	28 (15%)	7 (23%)	21 (13%)		
**Option for termination**	(missing 2)			0.664	
I will consider termination	79 (42%)	12 (40%)	67 (42%)		
I will not consider termination	17 (9%)	4 (13%)	13 (8.2%)		
I have doubts about termination	92 (49%)	14 (47%)	78 (49%)		
**Risk trisomy 21**	(missing 0)			0.528	
High (1/11–1/250)	23 (12%)	2 (6.5%)	21 (13.2%)		
Intermediate (1/251–1/1100)	149 (78%)	25 (81%)	124 (78%)		
Low (<1/1100)	18 (10%)	4 (13%)	14 (8.8%)		
**Risk trisomy 18**	(missing 0)			1.000	
High (1/11–1/250)	1 (1%)	0 (0%)	1 (0.6%)		
Intermediate (1/251–1/1100)	2 (1%)	0 (0%)	2 (1.3%)		
Low (<1/1100)	187 (98%)	31 (100%)	156 (98%)		
**Risk trisomy 13**	(missing 0)			1.000	
High (1/11–1/250)	1 (1%)	0 (0.00%)	1 (0.6%)		
Low (<1/1100)	189 (99%)	31 (100%)	158 (99%)		

**Table 2:** Women and pregnancy characteristics showing significant differences according to the couple’s option for pregnancy termination.

	I Will Consider Pregnancy Termination (N = 79)	I Will Not Consider Pregnancy Termination (N = 17)	I Have Doubts about Pregnancy Termination (N = 92)	Overall *p*
**Educational level**	**(missing 1)**			0.029
Up to secondary	6 (7.7%)	5 (29%)	13 (14%)	
Professional training	4 (5.1%)	3 (18%)	9 (9.8%)	
College	68 (87%)	9 (53%)	70 (76%)	
**Ethnicity**	**(missing 0)**			<0.001
European	69 (87%)	3 (18%)	64 (70%)	
Latin American	10 (13%)	12 (71%)	17 (19%)	
Asian	0 (0%)	1 (5.9%)	3 (3.3%)	
Other	0 (0%)	1 (5.9%)	8 (8.7%)	
**Previous cfDNA**	**(missing 2)**			0.004
No	62 (79%)	17 (100%)	86 (94%)	
Yes	17 (22%)	0 (0%)	6 (6.5%)	
**Sufficient information**	**(missing 1)**			0.002
No	2 (2.5%)	5 (29%)	10 (11%)	
Yes	77 (98%)	12 (71%)	82 (89%)	

**Table 3:** Items from the perspectives and satisfaction questionnaire (A) and Decisional Conflict Scale (B) showing significant differences between the target and the genome-wide groups.

	Total (N = 190)	Target (N = 31)	Genome-Wide (N = 159)	Overall *p*
**A. I feel I can manage an uncertain result well**	(missing 3)			0.009
Totally disagree	7 (3.7%)	3 (9.7%)	4 (2.6%)	
Disagree	33 (17%)	10 (32%)	23 (15%)	
Neither agree nor disagree	73 (38%)	10 (32%)	63 (40%)	
Agree	42 (22%)	2 (6.5%)	40 (26%)	
Totally agree	32 (17%)	6 (19%)	26 (17%)	
**A. The results of the test I have chosen will help me decide in my pregnancy**	(missing 2)			0.029
Totally disagree	7 (3.7%)	0 (0%)	7 (4.5%)	
Disagree	4 (2%)	2 (6.5%)	2 (1.3%)	
Neither agree nor disagree	21 (11%)	7 (23%)	14 (8.9%)	
Agree	47 (25%)	9 (29%)	38 (24%)	
Totally agree	109 (57%)	13 (42%)	96 (61%)	
**A. I want to know any information that may affect my child** **’** **s health, even in the future**	(missing 0)			<0.001
Totally disagree	3 (1.6%)	1 (3.2%)	2 (1.3%)	
Disagree	6 (3.2%)	3 (9.7%)	3 (1.9%)	
Neither agree nor disagree	15 (8%)	9 (29%)	6 (3.8%)	
Agree	27 (14%)	6 (19%)	21 (13%)	
Totally agree	139 (73%)	12 (39%)	127 (80%)	
**A. I want to know any information that may affect my child** **’** **s health, but not in the future**	(missing 5)			0.040
Totally disagree	46 (24%)	6 (21%)	40 (26%)	
Disagree	40 (21%)	3 (11%)	37 (24%)	
Neither agree nor disagree	21 (22%)	7 (25%)	14 (8.9%)	
Agree	28 (15%)	7 (25%)	21 (13%)	
Totally agree	50 (26%)	5 (18%)	45 (29%)	
**B. I am satisfied with the decision**	(missing 3)			0.036
Totally disagree	1 (0.5%)	1 (3.2%)	0 (0%)	
Neither agree nor disagree	5 (2.6%)	2 (6.5%)	3 (1.9%)	
Agree	59 (31%)	6 (19%)	53 (34%)	
Totally agree	122 (64%)	22 (71%)	100 (64%)	

## Data Availability

The data presented in this study are available on request from the corresponding author due to the inclusion of personal patient information.

## References

[1] Borrell A., Casals E., Fortuny A., Farre M.T., Gonce A., Sanchez A., Soler A., Cararach V., Vanrell J.A. (2004). First-trimester screening for trisomy 21 combining biochemistry and ultrasound at individually optimal gestational ages. An interventional study. Prenat. Diagn..

[2] Stokowski R., Wang E., White K., Batey A., Jacobsson B., Brar H., Balanarasimha M., Hollemon D., Sparks A., Nicolaides K. (2015). Clinical performance of non-invasive prenatal testing (NIPT) using targeted cell-free DNA analysis in maternal plasma with microarrays or next generation sequencing (NGS) is consistent across multiple controlled clinical studies. Prenat. Diagn..

[3] Pertile M.D., Flowers N., Vavrek D., Andrews D., Kalista T., Craig A., Deciu C., Duenwald S., Meier K., Bhatt S. (2021). Performance of a Paired-End Sequencing-Based Noninvasive Prenatal Screening Test in the Detection of Genome-Wide Fetal Chromosomal Anomalies. Clin. Chem..

[4] Van Der Meij K.R.M., Sistermans E.A., Macville M.V.E., Stevens S.J.C., Bax C.J., Bekker M.N., Bilardo C.M., Boon E.M.J., Boter M., Diderich K.E.M. (2019). TRIDENT-2: National Implementation of Genome-wide Non-invasive Prenatal Testing as a First-Tier Screening Test in the Netherlands. Am. J. Hum. Genet..

[5] Jani J.C., Gil M.M., Benachi A., Prefumo F., Kagan K.O., Tabor A., Bilardo C.M., Di Renzo G.C., Nicolaides K.H. (2020). Genome-wide cfDNA testing of maternal blood. Ultrasound Obstet. Gyne.

[6] Bekker M.N., Henneman L., Macville M.V.E., Sistermans E.A., Galjaard R.J.H. (2020). Benefit vs. potential harm of genome-wide prenatal cfDNA testing requires further investigation and should not be dismissed based on current data. Ultrasound Obstet. Gyne.

[7] Benítez-Quintanilla L., Pauta M., Matas I., Madrigal I., Borrell A. (2021). Cell-Free DNA Testing: What Is the Reason Why High-Risk Women Choose It?. Fetal Diagn. Ther..

[8] Generalitat de Catalunya, Agència de Salut Pública de Catalunya (2018). Protocol de Cribratge Prenatal D’Anomalies Congènites a Catalunya. https://salutpublica.gencat.cat/web/.content/minisite/aspcat/promocio_salut/embaras_part_puerperi/protocol_cribratge_prenatal/Protocol-cribatge-prenatal-anomalies-congenites-2018.pdf.

[9] O’Connor A.M. (1995). Validation of a Decisional Conflict Scale. Med. Decis. Mak..

[10] Garvelink M.M., Boland L., Klein K., Nguyen D.V., Menear M., Bekker H.L., Eden K.B., LeBlanc A., O’Connor A.M., Stacey D. (2019). Decisional Conflict Scale Use over 20 Years: The Anniversary Review. Med. Decis. Mak..

[11] McAllister M., Wood A., Dunn G., Shiloh S., Todd C. (2011). The Genetic Counseling Outcome Scale: A new patient-reported outcome measure for clinical genetics services. Clin. Genet..

[12] Guerrero-Peral Á.L., Porta-Etessam J., Rodríguez-Vico J., Núñez M., Ciudad A., Díaz-Cerezo S., Garí-Peris C., Pérez-Sádaba F.J., Lizán L., Santos-Lasaosa S. (2022). Adaptation and Validation of the Spanish Version of Decisional Conflict Scale in People with Migraine in Spain. Patient Prefer. Adherence.

[13] Calderon C., Ferrando P.J., Lorenzo-Seva U., Higuera O., Ramon Y Cajal T., Rogado J., Mut-Lloret M., Rodriguez-Capote A., Jara C., Jimenez-Fonseca P. (2019). Validity and Reliability of the Decision Regret Scale in Cancer Patients Receiving Adjuvant Chemotherapy. J. Pain Symptom Manag..

[14] Van Der Meij K.R.M., Van De Pol Q.Y.F., Bekker M.N., Martin L., Gitsels-van Der Wal J., Van Vliet-Lachotzki E.H., Weiss J.M., Galjaard R.-J.H., Sistermans E.A., Macville M.V.E. (2023). Experiences of pregnant women with genome-wide non-invasive prenatal testing in a national screening program. Eur. J. Hum. Genet..

[15] Lannoo L., Van Der Meij K.R.M., Bekker M.N., De Catte L., Deckers S., Devriendt K., Roggen N., Galjaard R.H., Gitsels-van Der Wal J., Macville M.V.E. (2023). A cross-country comparison of pregnant women’s decision-making and perspectives when opting for non-invasive prenatal testing in the Netherlands and Belgium. Prenat. Diagn..

[16] Bakkeren I.M., Henneman L., Van Vliet-Lachotzki E.H., Martin L., Gitsels-van Der Wal J.T., Polak M.G., Bekker M.N., Galjaard R.-J.H. (2024). Psychological impact of additional findings detected by genome-wide Non-Invasive Prenatal Testing (NIPT): TRIDENT-2 study. Eur. J. Hum. Genet..

[17] Hui L., Barclay J., Poulton A., Hutchinson B., Halliday J.L. (2018). Prenatal diagnosis and socioeconomic status in the non-invasive prenatal testing era: A population-based study. Aust. N. Z. J. Obstet. Gynaecol..

[18] Thorsen M.M., Khanuja K., Mahoney R.C., Al-Kouatly H.B., Russo M.L. (2024). Knowledge gaps and confidence in counseling about aneuploidy screening and testing: A survey of prenatal care clinicians. Prenat. Diagn..

[19] Van Den Bogaert K., Lannoo L., Brison N., Gatinois V., Baetens M., Blaumeiser B., Boemer F., Bourlard L., Bours V., De Leener A. (2021). Outcome of publicly funded nationwide first-tier noninvasive prenatal screening. Genet. Med..

